# The BridgeDb framework: standardized access to gene, protein and metabolite identifier mapping services

**DOI:** 10.1186/1471-2105-11-5

**Published:** 2010-01-04

**Authors:** Martijn P van Iersel, Alexander R Pico, Thomas Kelder, Jianjiong Gao, Isaac Ho, Kristina Hanspers, Bruce R Conklin, Chris T Evelo

**Affiliations:** 1 Department of Bioinformatics - BiGCaT, Maastricht University, Maastricht, the Netherlands; 2 Gladstone Institute of Cardiovascular Disease, San Francisco, CA 94158, USA; 3 Department of Computer Science, University of Missouri, Columbia, MO 65201, USA; 4 Departments of Medicine and Cellular and Molecular Pharmacology, University of California San Francisco CA 94143, USA

## Abstract

**Background:**

Many complementary solutions are available for the identifier mapping problem. This creates an opportunity for bioinformatics tool developers. Tools can be made to flexibly support multiple mapping services or mapping services could be combined to get broader coverage. This approach requires an interface layer between tools and mapping services.

**Results:**

Here we present BridgeDb, a software framework for gene, protein and metabolite identifier mapping. This framework provides a standardized interface layer through which bioinformatics tools can be connected to different identifier mapping services. This approach makes it easier for tool developers to support identifier mapping. Mapping services can be combined or merged to support multi-omics experiments or to integrate custom microarray annotations. BridgeDb provides its own ready-to-go mapping services, both in webservice and local database forms. However, the framework is intended for customization and adaptation to any identifier mapping service. BridgeDb has already been integrated into several bioinformatics applications.

**Conclusion:**

By uncoupling bioinformatics tools from mapping services, BridgeDb improves capability and flexibility of those tools. All described software is open source and available at http://www.bridgedb.org.

## Background

Many interesting problems in bioinformatics require the integration of experimental data from different sources. Examples include merging two independently created protein-protein interaction networks in Cytoscape[1] and visualizing microarray data on a collection of biological pathways in GenMAPP[2] or PathVisio[3]. More often than not, data of different types and from different sources are annotated with different identifiers. Thus, an important step in the analysis workflow is deducing which identifiers from one set correspond to which identifiers in the other set.

This problem of identifier mapping has been recognized, and a number of resources have been developed to solve it, including DICT[4], CRONOS[5], MatchMiner[6], AliasServer[7], PICR[8], Synergizer[9] and Ensembl BioMart[10]. For the most part, these resources accurately map identifiers and provide an interface, usually a web site, to the mappings. However, each resource necessarily has a focused domain of application based on limitations in resources and in the interest of its developers. Mapping services differ in aspects, such as coverage of species, coverage of identifier types, access speed and frequency of database updates. This has created two challenges for developers of bioinformatics applications. The first challenge is to develop software that is not tied to a single identifier mapping service. Tools that are built around a single service would have to be adapted with considerable effort if a more suitable service comes along. Optimally, switching should be a simple matter of configuration. The second challenge is to combine mapping services to get the benefits of each. For example, one could combine a small mapping table of probe identifiers of a custom microarray with a large mapping resource, such as Ensembl BioMart, or one could combine metabolite mappings and gene mappings when assessing experimental data from a combination of different omics platforms.

**Figure 1:**
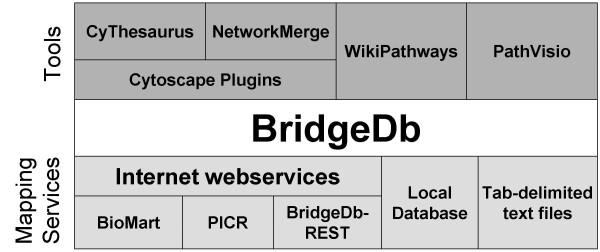
**The BridgeDb architecture**. BridgeDb provides a channel to connect multiple bioinformatics tools, such as Cytoscape or PathVisio, with online and offline identifier mapping services.

The key to both challenges is to create a standard interface between tools and mapping services. Here we present BridgeDb, a framework that provides such a standard interface. BridgeDb is a software library intended to be used by bioinformatics tool developers. The overall architecture is described in Figure 1. BridgeDb makes is possible to write shorter and simpler code to handle identifier mapping. This framework has already been incorporated in several bioinformatics applications.

### Concepts

To explain the features of the BridgeDb framework, it is necessary to define some concepts. We use the term **data source **to describe a database of biological entities, indexed by unique identifiers. Usually, these identifiers are assigned and maintained internally by an independent organization. Examples of data sources include Ensembl, UniProt, ChEBI, and the Gene Ontology.

An **internal identifier **is an identifier that is valid within the namespace of a data source. A good identifier must be unique, stable, and preferably free of semantic information. An internal identifier is not necessarily globally unique because a given identifier may be valid for two different data sources (this is especially the case for data sources that use simple integers as identifiers, such as Entrez Gene and PubChem).

In contrast with identifiers, we use the term **symbol **for a string that is a human readable representation of a biological entity. Symbols are not necessarily guaranteed to be unique or stable over time. A symbol can be a gene name such as INSR or TP53 for example. A biological entity might have several synonymous symbols or aliases. Since these symbols have a biological meaning, they are not semantic-free and do not serve as good identifiers. Semantics should be avoided because the information can be found to be "wrong" by new evidence. For example, the *Caenorhabditis elegans *gene symbol rad-5 (implying radiation sensitivity) was replaced by clk-2 (implying a function in developmental timing) after additional experimental evidence was collected and reinterpreted [11].

A **global identifier **is a globally unique identifier based on the combination of data source and internal identifier. Importantly, for the exchange of data, global identifiers must be standardized. For the representation of global identifiers, BridgeDb relies on the MIRIAM URI standard [12]. MIRIAM describes a minimal set of information to define a biological model and requires that biological identifiers be sufficiently descriptive. A valid MIRIAM URI contains both the data source and internal identifier (e.g., urn:miriam:uniprot:P62158).

We use the term **mapping service **to describe a resource for mapping information among identifiers from two or more data sources. This broad definition could include simple tables in a text file as well as complete relational databases or online web services such as Ensembl BioMart and PICR.

### Transitivity

**Figure 2:**
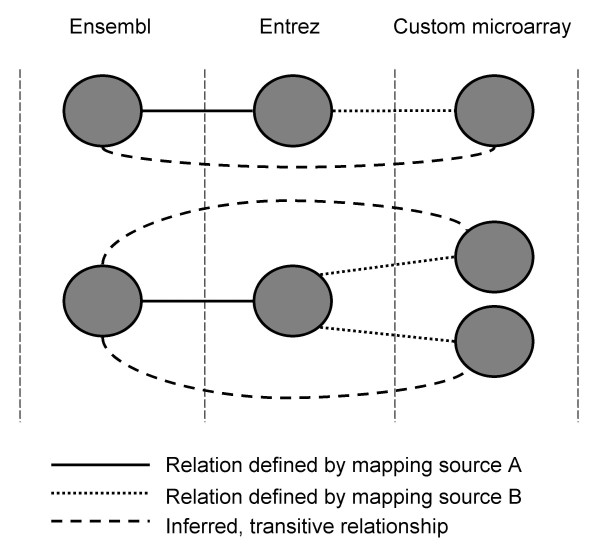
**Transitive relationships between identifiers**. In this diagram, two mapping sources (named A and B) are combined. Mapping source A defines relations between Ensembl and Entrez genes. Mapping source B is a custom microarray annotation that provides mappings between custom identifiers and Entrez. Through transitivity, the relation between the custom microarray and Ensembl can be inferred. The custom annotation file needs to define only a subset of relations.

BridgeDb allows stacking, or combining, of different mapping sources both in transitive and non-transitive modes. Transitivity means the inference of second- or even third-degree mappings from direct mappings. Transitivity becomes an important issue especially when combining mapping services in a stack. In general it is preferable to make use of global resources such as Ensembl BioMart for annotation of microarray data, but this is not possible for custom microarrays that are not distributed widely. In those cases, custom annotation files must be used. A custom annotation file can be produced to map custom identifiers to all other biological identifiers that one might wish to consider. But BridgeDb allows an alternative approach, where one only needs a very simple mapping of custom identifiers to one gene identifier, and can rely on Ensembl BioMart to provide the rest. For example, if the custom annotation file defines relations between custom identifiers and Entrez Gene, and Ensembl BioMart provides mapping between Ensembl and Entrez Gene, then mappings between the custom identifier and Ensembl can be inferred. In this way, the task of creating custom annotations is much simpler, while at the same time enabling broader coverage of data sources. This process is depicted in Figure 2. In the same way two specialized mapping services could be chained together. For example, PICR is specialized in proteins and is not normally capable of mapping gene identifiers. However, PICR can be combined with Ensembl BioMart, using the latter to translate gene identifiers into protein identifiers that the former can understand. In principle, transitive mapping from genes to transcripts to proteins could be achieved.

## Implementation

The implementation of BridgeDb is described here for each of the three layers of Figure 1: tools, interface and mapping services, starting with the interface.

### Implementation of the interface layer

The Application Programming Interface (API) of BridgeDb takes two different forms. The first form is based on Java and is suitable for Java applications. The other is based on Representational State Transfer (REST) and is suitable for all other programming languages.

#### BridgeDb Java API

**Figure 3:**
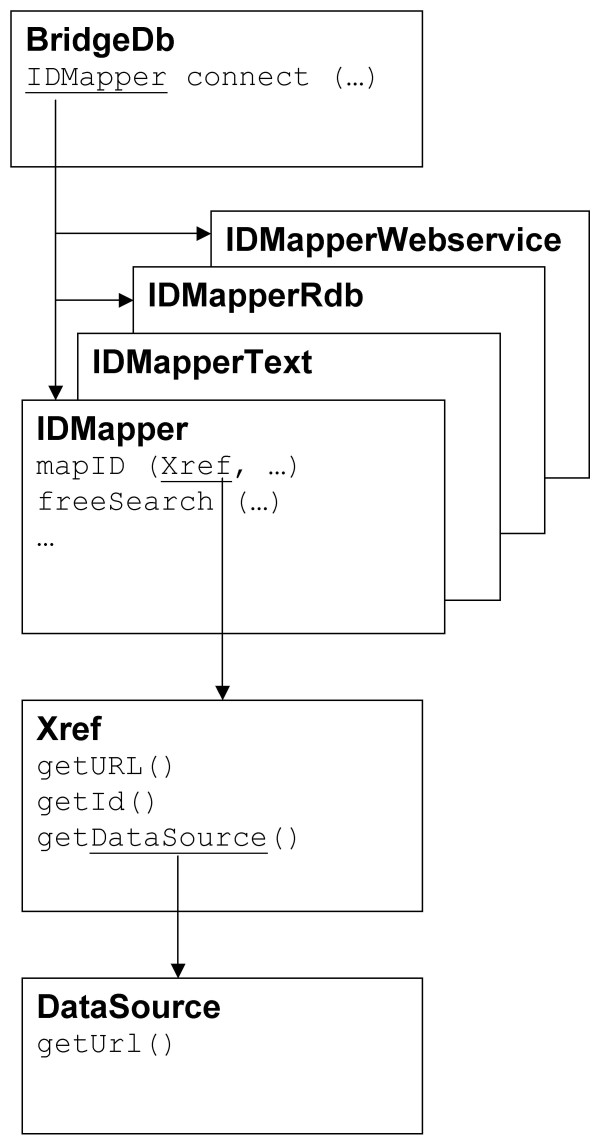
**Simplified UML Diagram of the BridgeDb java library**. The BridgeDb.connect method serves as an entry point, instantiating one of the many IDMapper implementations. The most important method of IDMapper is mapID, which takes an Xref object as argument. An Xref is a combination of an identifier String and a DataSource object, the latter representing an online biological database.

The structure of the Java API is represented in Figure 3. The central BridgeDb class keeps track of all available IDMapper implementations and provides uniform access to them through the static connect method. This method takes a connection string, which contains the requested mapping service and all parameters for configuring that service. To access a completely different mapping service, only the connection string has to be modified, the rest of the program can stay the same. The connect method returns an implementation of the IDMapper interface. This interface provides methods for mapping identifiers, as well as free text search and capability introspection. The mapID method of IDMapper performs the actual mapping job. It takes an Xref object as argument. Xref is a combination of a data source and a local identifier; these two are combined to form a global identifier. The Xref class also provides methods for generating valid web links to pages describing a biological entity, and for generating a valid MIRIAM URI. Data sources are themselves represented by DataSource objects, which also hold extra information such as web links to the main page of a data source. We have created a simple example in Java that shows how to map identifiers through webservices using BridgeDb (Additional file 1). To illustrate the usefulness of the standardized interface, we recreated the same functionality in a program without using BridgeDb (Additional file 2). In the latter case specialized code had to be written for each webservice, which makes the code more complex, and less flexible. For a full description of the API and more examples, see the developer documentation on the BridgeDb website [13].

#### BridgeREST API

In addition to the Java API, we provide a REST-based interface. The required software can run in the background and can be embedded in non-Java applications. Each function is accessed by a URL that specifies the address of the service and query parameters. For example, http://localhost/Human/search/ENSG00000122375 includes the following query parameters: 'search' specifies the type of query, 'Human' is the organism database being searched, and 'ENSG00000122375' is the query input. In this example, a list of identifiers and data sources corresponding to the query would be returned in the form of tab-delimited plain-text. Developer documentation for BridgeREST is also available on the BridgeDb website [14].

### Implementation of the mapping services layer

The mapping services supported by BridgeDb can be broadly categorized in three groups: flat files, relational databases, and online web services. Flat text files are great for custom annotations. Local relational databases provide the fastest access. They are stable and make long-running analyses repeatable. Web services are not as fast as local databases but potentially provide the broadest range of data and if well maintained, are more up-to-date.

In addition to supporting a number of third-party mapping services, BridgeDb also provides its own mapping services, in the form of BridgeDerby databases for efficient mapping of genes, proteins and metabolites, and in the form of a webservice named BridgeWebservice. Thus, BridgeDb can be used as a complete identifier mapping solution out-of-the-box for common bioinformatics applications. See Table 1 for a list of supported mapping services and by whom they are provided.

**Table 1:** Mapping services currently supported by BridgeDb

Description	Category	Provided by
Tab delimited text files	Flat file	Anybody
BridgeDerby for genes and proteins	Relational database	BridgeDb (Gladstone Institute)
BridgeDerby for metabolites	Relational database	BridgeDb (Gladstone Institute)
EnsMart	Web service	European Bioinformatics Institute
PICR	Web service	European Bioinformatics Institute
BridgeWebservice	Web service	BridgeDb (Gladstone Institute)
Synergizer	Web service	Harvard Medical School
CRONOS	Web service	Helmholtz Zentrum

The BridgeDerby mapping service is based on the Derby[15] relational database management system. Its advantage is that it can be used to create local databases that consist of just a single file. These files can be downloaded, copied and installed easily. This system, along with the database schema, has been described before[3]. The schema can be briefly summarized as follows. Each database consists of three tables. The DataNode table contains a list of local identifiers plus a short two-letter abbreviation for the data source. The Link table contains a list of relationships between identifiers. Each link has a left part and a right part; each part refers to a unique data source and identifier in the DataNode table. Finally, the Attribute table contains symbols and other attributes for DataNode entries.

We create database files per species for genes and proteins. We also created a database file for metabolites, which is species-independent. Using the stacking mechanism, the metabolite database and a species-specific gene and protein database can easily be combined to form a complete database for biological entities of a given species. The procedures for creating these two types of database files are described below in more detail.

#### Ensembl-based gene and protein database files

The database files for genes and proteins are based on Ensembl[16]. Relevant MySQL tables from Ensembl are locally installed and accessed via Ensembl's Perl API. Identifiers, annotations and cross-references are extracted, transformed and ultimately loaded into Apache Derby databases. The Derby databases are rebuilt and released after each Ensembl database release. Each Derby database contains information for a single species. Currently, databases are only produced for species of interest, including 36 different animal, plant, fungal, and bacterial species. However, Derby databases can readily be generated for any species supported by Ensembl or that has been effectively "ensemblized" (i.e., loaded into a database schema that is compatible with Ensembl's Perl API). Depending on the species, a selected subset of data sources are extracted from Ensembl and maintained in the Derby databases. Typically, these include Ensembl, Entrez Gene, UniProt, UniGene, RefSeq, miRBase, RFAM, PDB, TIGR, UCSC Genome Browser, and WikiGene, as well as a representative model organism database, such as MGI, WormBase, ZFIN, EcoGene or TAIR, and annotations from Gene Ontology, OMIM, BioGrid, Affymetrix, Agilent, and Illumina.

The same information as contained in the Derby databases is also stored in MySQL databases, using the same schema. This form of the data is maintained for web service accessibility. Practical limitations on the size of the distributed Derby databases are not an issue for MySQL. Thus, additional identifier systems, annotations and cross-references can be stored in the MySQL databases. Furthermore, with greater capacity, this system will be able to support additional types of information such as exon, transcript, and protein domain alignments, polymorphisms, and homology, which are also available from Ensembl.

#### HMDB-based metabolite database files

Currently, the database files for metabolites are based on HMDB Metabocards[17], because they provide free and easy-to-parse access to mapping information. Each Metabocard contains cross-references to CAS, ChEBI, PubChem and Kegg. Each metabocard is parsed by a script and identifiers are added to the DataNode table. Mappings between them are added to the Link table. This database assumes symmetry and transitivity (i.e., all identifiers on a Metabocard map to all other identifiers on the same card). The official name and all synonyms are stored in the Attribute table as symbols.

### Implementation of the tool layer

Broadly speaking, BridgeDb can be useful whenever an application needs to make use of multiple mapping services or wants to enable the user to choose among different services. To demonstrate this, we have integrated BridgeDb into a number of bioinformatics tools. These implementations are described below illustrating four different occurrences of the identifier mapping problem.

#### Use Case 1: Annotating biological pathways

*WikiPathways *is a collection of biological pathways open to community curation[18]. Pathways are networks of genes, proteins and metabolites that serve as a model for the actual biology of a cell. Pathway components must be properly annotated to maintain the consistency and integrity of the model for data mapping, updating and exchange. The common names of genes, proteins and metabolites are usually not suitable, because they can be ambiguous and can change over time. Identifiers from various data sources can provide exact and unambiguous references. BridgeDb is used by WikiPathways to provide integrated access to the most relevant identifiers by stacking species-specific databases for genes and proteins with a generic database for metabolites. The free search mechanism of BridgeDb helps curators find the correct identifiers for a broad range of biological entities. And BridgeDb also provides link-out URLs to all cross-references to help confirm the validity of an annotation and access more information from primary data sources.

#### Use Case 2: Merging biological networks

The *Network Merge *plugin for Cytoscape[1] can align, compare and merge networks. A common scenario for Network Merge includes two networks that represent overlapping biological components (e.g., protein-protein interaction networks from two different yeast two-hybrid experiments). There are many valid ways to annotate such networks, but to align, compare or merge them they must be annotated with identifiers from a single data source. To solve this problem, the Cytoscape Network Merge plugin utilized BridgeDb to unify identifiers of the biological entities when merging networks, so that overlaps among networks can be recognized.

#### Use Case 3: Mapping experimental data onto biological pathways

*PathVisio*[3] is a pathway visualization and analysis tool which has recently gained the capability to import genomics data and link it to pathways. To ease the process of importing biological datasets, BridgeDb is used to map microarray reporters to the corresponding genes and proteins. The standard Ensembl-derived BridgeDerby databases contain information about a number of common chip designs. Each identifier in the experimental data is mapped to an identifier in the pathway if possible, and if a match is found, that part of the pathway can be colored depending on the measured gene expression.

#### Use Case 4: Identifier translation

The *CyThesaurus *plugin for Cytoscape can perform large-scale identifier translation on biological entities in Cytoscape networks using BridgeDb. The plugin can be used for different purposes. For example, when multiple identifier sources are used in the networks of interest, this plugin can be used to translate different types of identifiers to a common identifier type so that identities of the biological entities in the networks will be unified. Alternatively, to export Cytoscape networks for use in other tools that require different identity types, one can utilize CyThesaurus plugin to translate the identifiers into identifier types that other tools can understand.

## Results and discussion

Once BridgeDb has been incorporated in a bioinformatics tool, it will be possible to choose a suitable identifier mapping solution for the job at hand. There are a few considerations when choosing the right service. First, when should two identifiers map to one another? What is the basis for a mapping? An identifier is a reference to a database record about a biological entity. If two databases describe the exact same biological entity, then certainly the identifiers should map to each other. But most applications require a broader definition of identifier mapping. When aligning microarray data with a pathway model based on genes, for example, there is a need for mapping microarray reporters to genes and gene products even though these are different biological entities.

The facile switching of mapping services makes it easy to compare them programatically. In a simple test we found that most web services integrated in BridgeDb are in agreement to a high degree. Four different webservices were able to map successfully more than 80% of a set of 1000 random Affymetrix probeset identifiers. For 72% of the total set, the result was identical for each webservice. For a given set of 100 Ensembl gene identifiers, each webservice was able to map over 91%, and the identical subset was 86% (See additional file 3). The source code of BridgeDb includes the script used for this comparison (See additional file 4). In short, webservices differ in coverage of species and identifier types, but when two webservices are both able to translate the same set of identifiers, they agree to a large degree.

A second issue that affects the choice of mapping service is a preference for local or remote access. The first option is more efficient in the case of high-intensity usage. Another advantage of a local system is that a source of data can be frozen to make analysis reproducible. A local system will not change in the middle of a long-running analysis procedure. Alternatively, the web service approach can be more up-to-date, is centrally managed and requires fewer resources (disk space) from the end-user.

Theoretically, it would be good to do away with the multiplicity of biological databases and designate a single universal identifier for each biological entity. However, this would not eliminate the need for identifier mapping, as there would still be a need to relate different biological entities such as transcripts and reporters. That fact, combined with the current situation of multiple gene databases, means that we have to deal with the identifier mapping problem in the best possible way. We believe that standardizing on a framework used by several programs in unison provides a robust and extensible solution.

Importantly, identifier mapping should be performed as late as possible in a given workflow or annotation scheme. Gene databases change over time as more information becomes available. Experimental datasets, on the other hand, are fixed once the experiment is done. The experimental data should be annotated as closely as possible to the experimental conditions. Consider for example microarray data. Perez-Iratxeta et al. [19] have found changes to 5% of reporter annotations over a two-year timespan. The mapping of reporters to genes is not static, and the data should be linked directly to the reporter sequence that was measured rather than to a gene or genomic location. Replacing probe identifiers with gene identifiers in a microarray dataset would limit future analyses to potentially outdated associations. The importance of BridgeDb in this context is that experimental data can be annotated and linked in a consistent manner over time, ensuring that the integrity of the data is maintained while the analysis utilizes the most up-to-date information about genes and gene associations.

Several mapping services supported by BridgeDb are provided by us (Table 1). We consider these good default choices that are applicable to a broad range of situations. The local databases for genes and proteins provided by BridgeDb are based on Ensembl. We find that Ensembl defines reliable homology-based mappings that are frequently updated and available for a large number of species and, thus, provides a reasonable default choice. However, some authors [5] have noted that high thresholds of sequence similarity lead to a failure to detect all correct mappings, so extra information derived from gene nomenclature should be used. The BridgeDb interface does not dictate what constitutes a correct mapping; this is determined by the underlying mapping services. The flexible architecture of BridgeDb makes it possible to switch to a service that employs a different basis for identifier mapping if desired.

The BridgeDb concept derived from our experience as developers of different bioinformatics tools. The Application Programming Interface (API) was designed to be used by multiple tools and has proven its usability for a combination of applications with different uses.

We believe that it is better to build identifier mapping into a tool rather than requiring users to perform identifier mapping manually or with separate tools. Burdening the researcher to perform identifier mapping ignores the problem and dramatically limits the usability of the tool. Relying on external solutions also introduces unknown factors into the workflow that can lead to unreliable analysis results. By integrating a mapping service directly in the bioinformatics software tools, error-prone data preparation steps are avoided.

Different software packages solve the identifier mapping problem in different ways. We propose to modularize the identifier mapping problem into a single library. This has several advantages. Using a shared library means that developers can pool efforts, rather than investing considerable effort into maintaining isolated solutions. Tools that currently do not implement identifier mapping could do so with very little effort by just adding a module to the project.

## Conclusions

BridgeDb frees bioinformatics tools from compromising on solutions to the identifier mapping problem. By providing a standardized layer through which different mapping services can be used, BridgeDb makes it easy for tool developers to support and switch between multiple services. BridgeDb can also be used to combine or merge mapping services to support multi-omics experiments or integrate custom resources.

## Availability & requirements

**Project name: **BridgeDb.

**Project home page: **http://www.bridgedb.org.

**Operating systems: **platform independent

**Programming language: **implemented in Java, compatible with any programming language.

**Other requirements: **Java Runtime Environment version 1.5 or higher.

**License: **Apache 2.0 License. Source can be found at http://svn.bigcat.unimaas.nl/bridgedb. A snapshot of the code is available as additional file 4.

**Any restrictions to use by non-academics: **none.

## Authors' contributions

MI drafted the paper. TK developed the first derby databases. AP developed the Ensembl ETL process for MySQL and derby database production. MI and JG designed the BridgeDb API. IH improved the Ensembl ETL process and developed the REST web service. JG developed both Cytoscape plugins. AP and KH contributed to the original GenMAPP identifier mapping strategy. CE and BC provided valuable feedback and support. All authors have read and approved the final manuscript.

## Supplementary Material

Additional file 1 **BridgeDb example in Java**. This is a simple example script that demonstrates how the BridgeDb library can be used to map an identifier using two different mapping services. Click here for file

Additional file 2 **Counter-example not using BridgeDb**. This is a counter-example that does the same as additional file 1, without using BridgeDb. Because specialized code has to be written for each webservice, this script is more complex and less flexible. Click here for file

Additional file 3 **Raw results of the comparison of four different webservices**. The script used to extract this data is included in additional file 4. Click here for file

Additional file 4 **Source code of the BridgeDb java library version 0.9**. For the latest version we recommend to use our subversion repository at http://svn.bigcat.unimaas.nl/bridgedb. Click here for file
